# Work-life boundary management of peer support workers when engaging in virtual mental health support during the COVID-19 pandemic: a qualitative case study

**DOI:** 10.1186/s12889-023-16488-9

**Published:** 2023-08-25

**Authors:** Elmira Mirbahaeddin, Samia Chreim

**Affiliations:** Telfer School of Management, University of Ottawa, 55 Laurier Ave E, Ottawa, Ontario K1N 6N5 Canada

**Keywords:** Work-life, Work-home, Boundary, Boundary management, Virtual work, Roles, Peer support, Mental health, COVID-19 pandemic

## Abstract

**Background:**

Mental health care needs have increased since the COVID-19 pandemic was declared. Peer support workers (PSWs) and the organizations that employ them have strived to provide services to meet increasing needs. During pandemic lockdowns in Ontario, Canada, these services moved online and were provided by PSWs from their homes. There is paucity of research that examines how providing mental health support by employees working from home influences their work-life boundaries. This research closes the gap by examining experiences of work-life boundary challenges and boundary management strategies of PSWs.

**Methods:**

A qualitative case study approach was adopted. Interviews with PSWs who held formal, paid positions in a peer support organization were conducted. Data was analyzed thematically using both inductive and deductive approaches. Descriptive coding that closely utilized participants’ words was followed by inferential coding that grouped related themes into conceptual categories informed by boundary theory. Member checking was conducted.

**Results:**

PSWs provided accounts of work-life boundary challenges that we grouped into three categories: temporal (work schedule encroachments, continuous online presence), physical (minimal workspace segregation, co-presence of household members and pets) and task-related (intersecting work-home activities). Strategies used by PSWs to manage the boundaries consisted of segmenting the work-life domains by creating separate timescapes, spaces and tasks; and integrating domains by allowing some permeability between the areas of work and life.

**Conclusion:**

The findings from this study can help inform management, practices, future research and policy on health care workforce. The study highlights the need to attend to the consequences of greater work-life integration for mental health workers since their successful practice is largely dependent on maintaining self-care. Training regarding work-life boundary management is highlighted as one of the ways to approach situations where work from home is required.

## Background

The COVID-19 pandemic has exacerbated mental health problems globally [1]. According to the World Health Organization, national surveys show a substantial increase in the prevalence of psychological distress in populations during the pandemic [2]. People have experienced aggravated mental health problems including major depressive disorder, anxiety, stress and posttraumatic stress disorder among others [3, 4]. While there has been an increase in mental health problems, mental health care capacity has not kept up with the demand due to difficulty in rapid adaptation to virtual care, high levels of burnout and absenteeism among health care workers, and prioritization of management of outbreaks among other reasons [5–9].

Mental health peer support services have been a viable resource during the pandemic [10] as peer support workers and the organizations that employ them have strived to maintain services to meet the increasing needs. Peer support workers (PSWs) – not to be confused with personal support workers— “are an integral part of the mental health workforce” ([11] p. 9); they are people who have lived experience of mental health issues and recovery and can mobilize their lived experience to provide support to others who are struggling with a range of mental health difficulties [12, 13]. Peer supporters may engage in voluntary peer support or – as in the focus of this paper – hold formal, paid positions in mental health/social services organizations [14].

In a number of jurisdictions, peer support transitioned from in-person to virtual services during the COVID-19 pandemic, often spurred by lockdowns (in the US [11]; in China [15]; in Brazil [16] and in the UK [17]). PSWs have been able to provide peer support from their homes, enabled by telecommunication tools [10]. However, for any occupation, working from home can involve challenges that include digital access and literacy, lack of workspace, and navigating work and home roles that could become entangled and cause work-family conflict [18–20].

There is a paucity of research that examines how providing virtual mental health support services by PSWs working from home influences the PSWs’ work-home boundaries. Most research that addresses PSWs’ work role boundaries tends to focus on how borders are established with peers (clients), and attends to issues of distance from peers, self-disclosure and after-hours involvement [21, 22]. The blurring of work-home boundaries and its impact on PSWs’ well-being has not been investigated, and neither has there been sufficient attention to how PSWs manage boundary issues. In this paper, we close these gaps by examining the experiences of PSWs who provided virtual mental health peer support from home during the COVID-19 pandemic. The purpose of the study is to examine what boundary challenges PSWs faced and how they managed boundaries. This is an important topic because blurred boundaries between work and home domains can be a source of strain and conflict [23–25], and yet peer support necessitates self-care and taking time to disconnect from work [26, 27].

Understanding how blurred home-life boundaries are experienced and managed is an important topic not only as it pertains to PSWs, but also as it may apply to a variety of health care workers. This is especially the case given the push to offer health services remotely in order to improve access, and given that these services may be provided from health care workers’ homes. The findings from this study can help inform management, practices, future research and policy on the health care workforce.

We conducted our study in a peer support organization operating in a large city in Ontario, Canada. We adopted a qualitative case study approach to explore in depth how PSWs experienced the work-home boundaries after the transition to virtual peer support, and how they managed the challenges they experienced. In the next section, we consider studies that have addressed the opportunities and challenges of virtual peer support, and briefly review research on home-life boundaries and boundary management.

### Opportunities and challenges of virtual peer support

Research on previous infectious disease outbreaks and health system approaches to managing the consequent mental health impacts shows that supportive community-based programs (such as routine peer support, psychological art programs and psychological first aid within communities) were effective in enhancing the response capacity of mental health systems [28]. Many of these community-based services, including peer support, have been offered through telehealth using different communication methods during COVID-19 as in-person meetings were severely restricted [29, 30]. Research conducted during the early stages of the COVID-19 restrictions documented that people in isolation enthusiastically sought virtual support to address mental health needs, showing a population interest and acceptance of this method of support service delivery [10, 30]. Systematic reviews recognize the utility of online peer support for various mental health and well-being services, for instance, internet-based peer support for parents [31] and peer support through mobile applications for distress alleviation [32] as well as different age groups including adolescents and young adults with mental health issues [33–35].

Despite the need for and public openness to virtual peer support, there has been limited research on the challenges to PSWs posed by virtual service delivery from home. A recent study on the impact of COVID-19 on PSWs by Adams et al [11] investigated how the pandemic affected PSWs’ day-to-day work, among other topics. The findings pointed to PSWs’ engaging in new job tasks such as learning about new technology, providing support remotely and facilitating online groups. PSWs who participated in the study reported high satisfaction with supervisory and organizational support and pointed to the benefits or positive impacts from the pandemic [11]. However, this study did not focus specifically on PSWs’ experiences of working from home. Studies on other health care workers show that working from home introduces disruption to the boundaries between the professional and personal lives of service providers. For example, a study on social workers reported challenges associated with maintaining work-life balance and blurring of work-life boundaries [36]. Rapp et al [37] explored the “work-nonwork boundaries” of healthcare workers during the pandemic and established the connection between the violation of work-nonwork boundary and the workers’ experience of burnout (exhaustion, detachment, and inefficacy). Overall, research shows that stress induced by a work-life imbalance negatively affected efficiency and decision-making ability leading to suboptimal care and poor productivity both at home and at work [38]. Thus, the inability to maintain work-life boundaries and balance has ramifications for the quality of life, work and client service of health care workers.

### Work-home/life boundaries and boundary management

Researchers have emphasized the distinctions between the work domain and other life domains (including the family and the home), using the term “boundaries” to talk about these distinctions (e.g. work-family or work-home boundaries) [23, 24, 39, 40]. Boundaries are created by people to simplify and classify the world around them and thus they have psychological and behavioral ramifications [39]. Boundaries “delimit the perimeter and scope of a given domain” such as a role, a home and a workplace ([40], p. 705). Roles provide specificity as to who an individual is or what the individual does in a given domain such as home or work [39]. The home role and the work role may be differentiated according to time, space and tasks, so an individual moving from one of these roles to the other typically crosses temporal, physical and task boundaries. Blurring of work-life domains has been shown to facilitate boundary crossings and enable the achievement of some goals [41, 42]. However blurred work-life boundaries can also have negative emotional impacts, compromise wellbeing and contribute to work-life conflict [41, 43, 44].

Boundary management refers to the tactics or strategies that individuals use to manage the intersection between work and non-work domains [23] Different boundary management tactics have been documented in the literature. One way to view boundary management is to consider the degree to which individuals segment or integrate domains. A review of the literature on work-family boundary management indicated that segmenting work and life roles was associated with lower conflict and better work-life balance [23]. Perception of work-life balance is positively associated with mental health [45]. Thus, while integrating and allowing permeability between the domains can lead to positive spillovers such that one role enriches the other, integration can also lead to negative spillovers such as role conflict and drain of one’s resources [24, 46].

Another way to view boundary management is to consider the type of boundary that is being managed. For example, in their empirical study of boundary management tactics, Kreiner et al [40] referred to temporal, physical, behavioral and communicative tactics. According to these authors, temporal tactics include removing oneself from work with the purpose of rest, e.g., taking a vacation. Physical tactics are related to adapting physical boundaries by establishing or removing physical borders between work and home. Behavioural tactics include using technology to facilitate boundary work, using other people and prioritizing important work and home demands. Communicative tactics include such practices as setting expectations regarding boundaries [40].

Researchers have referred to “idiosyncratic” approaches to the management of boundaries that may be adopted by individuals [39, 40]. It is also understood that there is variation in the degree of control that individuals may exert over boundaries [24]. Hence, boundary management varies by individual (e.g. according to preferences) and by situation and context (e.g. according to family obligations).

There have been empirical studies on boundaries and boundary management in different contexts, but to our knowledge, none have been conducted on the experiences of PSWs or other mental health workers during the pandemic. Hence, we ask the following research questions: What work-home boundary challenges arose for PSWs when working virtually from home during the COVID-19 pandemic? How did PSWs manage the work-home boundary challenges? We focus specifically on the work-home boundary, and we use this term interchangeably with work-life boundaries in our findings. We focus on work-home boundaries because the context of our study was one of imposed lockdowns and stay-at-home/work-from-home mandates.

## Methods

### Aim and research approach

The aim of this study was to examine the work-home boundary challenges PSWs experienced and their boundary management tactics. We adopted a qualitative case study research approach as it provides an in-depth understanding of people’s experiences and the context surrounding these experiences [47]). The strengths of qualitative methods include the ability to pay strong attention to context, and respect and report on the experience of participants [47, 48]. Our approach allowed achieving a deep understanding of workers’ experiences and management of work-home boundaries.

### Setting and context

We conducted our study with members of a peer support organization that operates in a large city in Ontario, Canada. Prior to the COVID-19 pandemic, the organization provided almost all its services in person in various programs, reaching a large peer (client) base. At the time of the study, the organization employed twenty-one paid PSWs, some of whom held managerial positions in the organization. The study was conducted during the earlier days of the pandemic when work-from-home mandates had been enacted in Ontario. We selected this case because the organization transitioned all its services to virtual platforms in a short period of time in order to meet the increasing demand for peer support at a time of lockdowns and isolation. This case allowed us to capture the richness and complexity of the emerging work-home boundary issues during the pandemic through the lens of PSWs’ experiences. The case is also illustrative of work-life challenges that individuals encounter during an enforced and very quick transition to work from home.

### Data collection

Semi-structured one-on-one interviews were conducted during the pandemic (February to June 2021) with paid PSWs, some of whom also held managerial positions in the organization. All paid PSWs of the organization were invited to participate in the study. Thirteen individuals (including four PSWs who held managerial positions) agreed to participate. This constituted 62% of paid members of the organization. Participants included 11 women and two men, most of whom held full-time positions. All participants were asked to describe their work roles prior to and post the work-from-home mandate. They were asked to describe their lived experiences of work-life boundary issues (opportunities and challenges) that occurred as they transitioned to providing virtual peer support. Further questions were asked about strategies they used to manage the work-home boundaries. Participants were encouraged to provide examples throughout. Interviews, which lasted between 60 and 90 minutes, were transcribed verbatim and anonymized.

### Data analysis

N-Vivo software was used to facilitate data coding and retrieval. The interviews were analyzed thematically (as outlined by Miles et al [47] and Hennink et al [49]) using both an inductive approach that captures the perspective of the participants and a deductive approach that is informed by theoretical notions. The constant comparison method was used to derive and refine themes across interviews. In the first stage of the analysis, descriptive coding that closely utilizes participants’ words was used [47]. This was followed by inferential (second-cycle) coding which allows the joining of related themes into a smaller set of categories that are more abstract [47]. At this stage we referred to the literature and used conceptual notions derived from boundary theory to aggregate the findings.

### Confirming findings: quality, credibility and trustworthiness

In November 2022, we conducted “member checking” [47] to collect feedback from participants and to confirm that our interpretations matched their experiences. This tactic is important in qualitative research on lived experiences, since as Miles et al [47] point out, participants in the setting are “bound to know more than the researcher ever will about the realities under investigation [and] can act as judges, evaluating the major findings of a study” ([47] p. 303). To ensure the credibility of our findings, we met with and presented our results to a senior manager and three PSW representatives of various services provided by the organization (e.g. hospital peer support, recreational peer support), and asked them for their feedback. We also sent all employee members of the organization the full draft of our paper and invited them to provide feedback on the paper. In total, we obtained feedback from seven organizational members. The feedback we received indicated that our results reflected the experiences of PSWs, but also offered refinements to our analysis. With respect to feedback on the Results, we were told that *“the results are spot on”* and *“the study really captured what I have been experiencing as a peer supporter working virtually”* but that some of the challenges we reported did not apply to a few of the PSWs who had had previous experience working virtually in a different context, or who had become proficient in setting boundaries over their many years of experience doing peer support. Hence, we included these topics in our Results section below. Participants also emphasized the importance of highlighting the sudden and radical nature of the change in work arrangement during the pandemic lockdown, which partly explains the many challenges we reported. We thus included this topic in the Discussion section.

## Results

We report our findings in three sections. In the first section, we focus on PSWs’ experiences of work-home boundary challenges, and in the second section, we elaborate on the boundary management tactics utilized by the PSWs. Table 1 summarizes the boundary challenges and boundary management tactics.

**Table 1:** Work-home boundary challenges and management tactics

**Work-home Boundary Challenges**	**Temporal Boundaries**	Work schedule encroachments
		Continuous online presence
	**Physical Boundaries**	Minimal workspace segregation
		Co-presence of household members and pets
	**Task Boundaries**	Intersecting work-home activities and tasks
**Work-home Boundary Management Tactics**	**Segmenting domains: closing borders and separating work from home:**	Negotiating and finding workarounds
	**Temporal tactics**	Actively limiting time spent on work activities
	**Segmenting domains: closing borders and separating work from home:**	Designating separate physical spaces for work and non-work activities
	**Physical tactics**	Using physical markings and objects to symbolically separate the domains
	**Integrating domains:**	Taking work-related calls during personal time
	**Opening borders and allowing some boundary permeability**	Addressing home-related topics in the context of work activities

It is worth noting that PSWs told us about opportunities that arose due to working from home, such as the ability to save time and money by not commuting to work every day, engaging in self-care such as cooking and eating lunch at home, and spending time with loved ones during work breaks. However, their accounts concentrated mostly on the challenges, and this is the focus of the paper. Attention to the challenges allows us to understand how PSWs exercised agency and insight in overcoming difficulties through boundary management, which we report in the second subsection. It also allows consideration of policies and organizational practices that can help mitigate the challenges (addressed in the Discussion).

### Experiences of work-home boundary challenges

Participants pointed to major challenges that we grouped into three categories: temporal work-home boundary challenges, physical work-home boundary challenges, and task boundary challenges. Temporal challenges address difficulties associated with intermingling work and non-work schedules, as well as what participants experienced as continuous online presence for work purposes, even during non-work hours. Physical challenges refer to difficulties separating home space/objects, and household members from work situations. Task challenges manifested in the intersection of home activities with work activities. We address each of these challenges next.

### Temporal work-home boundary challenges

Temporal work-life boundary challenges consist of work schedule encroachments and continuous online presence.

#### Work schedule encroachments

Early in the pandemic, worksite (office, hospital) peer support services were cancelled, and all the services shifted to online and phone support. Thus, the work schedule of the PSWs had to change to adapt to the new way of providing services in virtual space. During the pandemic, PSWs experienced constant modifications to their work schedules because of uncertainties associated with unforeseeable rules relating to COVID-19 restrictions and because achieving a better understanding of peer needs led to changes in programs offered. These changes caused less predictability and less ability to plan for non-work activities:

*“It’s been very unstable, we’re constantly changing to try to meet whatever the government is doing and what the peers want: updating programming, finding new ways to do things. I had a very predictable schedule … , but now … we’re moving things around … which makes things difficult.”*

The work schedule challenges were related not only to instability and unpredictability but also to difficulty segmenting and sequencing work and life activities. Before the pandemic, PSWs had clear distinctions between work and non-work hours, and most typically had one block of several hours a day dedicated to working, and these timescapes allowed PSWs to segment work activities from other life activities. However, during the pandemic, the peer support tasks were distributed in split blocks of time with non-work intervals throughout the day:

*“I would drop my kids off at school, I got to work, and would park my car a little bit away and walk to work, so I have a little time for myself … and then [at the end of the workday] the whole thing in reverse, walk back to my car... Whereas at home, I may have a group in the afternoon on zoom and then another group later in the day... It’s all disjointed and, in between, I don’t have work to do, but I don’t feel like I’m really relaxed.”*

In addition to work scheduling being disjointed, PSWs reported an increase in work owing to increased need among peers and coworkers. A PSW stated: *“My workload is so heavy that I’ve run a to-do list that’s several pages long every day. By the time I get through that, I have to write another one, so it’s like the workload never ends.”* PSWs faced difficult situations when they had to set boundaries: *“I sometimes don’t understand what’s an unreasonable ask … I’m trying to suss out needs and that for me is probably one of my biggest weaknesses. I just sometimes don’t know how to set the right boundaries”.* People’s expectations about PSWs’ responsiveness and accessibility were challenging to manage. A PSW explained that:

*“Boundaries around time are really interesting because some people [peers] expect you to be available Monday to Friday 9 to 5, no questions asked. Some people are expecting you to be available before or after that … They’ll send you an email saying ‘this is what I need’ – and it’s really hard to set boundaries.”*

Moreover, some PSWs’ sense of responsibility and solidarity with peers or coworkers overrode their need to set boundaries. They felt compelled to respond to requests outside of their work hours even at the expense of their well-being. A PSW spoke about feeling “*a sense of responsibility”* towards coworkers given that there was *“a lot of burnout and if someone needs me, I want to be there for them.”* Another PSW stated:

*“I have a really hard time telling people no if they say, ‘I need to speak with you’ … I’m still really trying to navigate the best way to have firm boundaries that are respectful of others... I’m always worried that things are gonna get dropped, that someone’s gonna get forgotten about or some piece is not gonna be picked up.”*

Before the pandemic, PSWs were able to transition from work to non-work and vice versa due to a time lag. During the pandemic, the lack of temporal separation between work and non-work, and the increased workload and expectations led to the blurring of work and non-work domains. What used to be separate timescapes with clear boundaries were now intertwined, with work often encroaching on non-work time.

#### Continuous online presence

When peer support services transitioned to a virtual space, several means of communication and connection became available, however, conventions of when and how these means could be accessed were not clear. Ease of access to work emails, chats, or phone calls furthered working longer hours. While before the pandemic the PSWs could check emails, they were now more tempted to answer an email when they were not on formal work time. Various reasons for increased engagement with work were identified. PSWs reported spending more time in front of a screen compared to pre-pandemic times. Being online was not necessarily for work reasons; nevertheless, many felt that by being online they were drawn to engaging in virtual peer support work. Finally, during the pandemic, PSWs connected more with social media and other resources online to obtain COVID-19 news.

*“I think COVID is providing a platform where we have our laptops and phones open all the time because we want to know what's happening outside our house. Especially when there's a lockdown, it's like, we're not able to go here, go there. So we're maybe looking for friends, what are they doing? What are other communities doing? So there's more of that inclination of seeing what's happening around the world.*

During this time, work-related emails were often present and open on screens and could hold PSWs engaged with work beyond work hours. Several participants indicated that they engaged in virtual non-work activities before the pandemic. However, during the pandemic, those non-work activities involved online presence where work activities also took place:

*“I play a lot of video games, but my work email is open now a lot of the time, so even if I’m off work, if I’ve forgotten to close my email, I’m looking at what’s going on at work when I’m usually pretty good in person at making sure that doesn’t happen. I have pretty firm boundaries that I don’t do work when I’m not working. I’ve lost a lot of that since we’ve been working from home because it always feels like there’s something going on.”*

For some PSWs, it would take effort to refrain from reading and replying to work-related messages during non-work hours when hearing an email notification sound. A PSW mentioned, “*it [the message] is constantly at the back of my mind”* commanding attention even during personal time. Another PSW spoke of difficulty “turn [ing] off the work mind”, and a third declared: “ … *if I hear an email coming in at nine o’clock at night, I’m going to look at it and I’m going to maybe respond, or it will be on my mind. So my time management with that can be a little tricky.*” In some situations, PSW felt a sense of responsibility to manage what was occurring online during off-work hours, especially because the pandemic led to the heightened use of online platforms that posed additional challenges:

*“We have that Facebook group, virtual drop-in group. So people can go on and make comments any time of the day or night. So when people were being inappropriate … here you are at 8:30 at night in your PJs. And you’re having to deal with the situation because you can’t leave it till the next morning, it’s on Facebook.”*

Thus, work-schedule encroachments and continuous online presence gave rise to heightened temporal boundary challenges.

### Physical work-home boundary challenges

As PSWs transitioned to working from home, the coexistence of work and home life led to the blurring of physical boundaries that previously delineated these domains. This section delves into two primary aspects: minimal segregation between the workspace and the home space, and the presence of household members and pets in what became the workspace during the pandemic.

#### Minimal workspace segregation

The early stages of the transition to working from home were described as “chaotic” and the PSWs found themselves in a space that was at once work and home. The co-presence of work and home life led to the blurring of the physical boundaries that were used to separate these domains. This was particularly challenging for PSWs who had limited discretionary space at home that they could customize as a separate workstation. A PSW who did not have an office set-up at home spoke about the challenge of “*having all work in the home”: “If I’ve had a stressful day at work, that lingers with me and I’m like ‘Ugh there’s my work piled up there and I don’t want to think about it.’”* Another indicated that having little space at home where work and sleep occur in one place leads to blurred physical boundaries. Before the pandemic, PSWs worked at hospitals or their offices, and they would leave behind their notebooks, documents, and work laptops at work sites. However, during the pandemic, these work-related objects were transferred to their home space (work-to-home transfer). Work objects now physically inhabited the home space, encroaching on home life. A PSW stated:

“*I set up [a workstation] in my [leisure] room, but I found I couldn’t divide the space. I would go in there and it was work, and before COVID it was my leisure space. I stopped really using it as that because it just felt too ‘work’.”*

There were home objects that now had to be used for work, such as desks, electronic equipment and others (home-to-work transfer), and this tended to be disruptive. A PSW stated: “*It’s chaotic, like ‘Okay now we’re going to eat dinner, clear everything’ to ‘Oh, I’m doing work now, bring it back’!”* Hence for many participants, working from home was disruptive to home life. Some PSWs took longer to adjust to the change, indicating that it was challenging to make a mental shift from being at work to being at home given the permeable boundaries:

*“I couldn't figure out how to make it work, how to be organized, and how to keep it separate. And it didn't even occur to me that I should make a separate workspace. I just was not in a good space. I was working at the dining room table or on the couch, wherever I could just take my laptop … And I would make notes from staff meetings and then I would lose the notes. I just could not seem to mentally make that shift.”*

#### Presence of family members and pets

Several PSWs commented on the co-presence of individuals from different domains in the space where work activities were undertaken. While on virtual (Zoom) meetings with peers or co-workers, household members or pets could be present in the room. A PSW stated: *“I was working … but then life was going on around me and I found that very distracting and frustrating*”, referring to the presence and activities of household members. Another PSW spoke about the experience of “immediacy” in sensing a household member’s physical presence when performing PSW work:

*“At first it was definitely really hard for me to focus … If I’m physically at work, I’m very focused on what I’m doing because I’m ‘at work’... There are so many distractions at home like that immediacy in knowing someone physically, the feeling of them being in the room, which I can’t express very well.”*

The quote above expresses discomfort in experiencing in the same space the presence of two domains – work and home – that until then had been separated physically. Individuals were not the only source of distractions. Pets could also command attention and pose challenges.

*“I have pets, so there’s been a lot of me being like: ‘Just one moment I need to go chase my dog” or ‘my dog is barking at something, and I need to check what the freaking out is about.’ … where that’s not something I’ve ever had to deal with before in a workspace. It also applies when I’m on the phone because my dog barks, so people will be like ‘Hey what’s your dog’s name, how long have you had your dog?’ … It’s more the psychological thing, like my own feelings about my space.”*

As the above indicates, the movement of work to the home space posed challenges in terms of PSWs being constantly reminded of the presence of work through work objects, inability to physically separate the two domains when one inhabited a small space, disruptions to home life because of use of home objects for work, and having to deal with the presence of household members and pets during work activities.

### Task boundary challenges

Work and home tasks and activities became intertwined, creating challenges for PSWs as we show below.

#### Intersecting work-home tasks and activities

In the above sections, we discussed the challenges associated with temporal and physical boundary permeability. There are implicit references in those sections to the challenges associated with enacting both work and home roles in the same space and same time. In this section, we further elaborate on the permeability of work and home roles that had been mostly segmented before the pandemic, giving attention to the experience of the intersection of the home and work tasks and activities for many PSWs. In the following PSW quote, the notion of role is captured by the notion of hat:

*“We talk about hats a lot in peer support, like I have my peer supporter hat, I’m wearing my friend hat, and I’ll be interchanging them. And that is a lot harder here. It feels like I’m home Jane, but at home Jane is taking care of her animals, at the same time peer support Jane is trying to take care of a group! Then it gets a little bit tangled.”*

PSWs who had younger children or children with special needs spoke at length about the experience of wearing different hats at the same time. With the pandemic lockdowns, schools and other facilities shut down. Schools moved classes online, and this often required that parents be available for their children’s classes online. This created a major burden on PSWs who needed to navigate the needs of their children and the work requirements:

*“It’s not like I could go separate myself. My kid is young, she’s 6, and she needed me around to help her with her schoolwork. She couldn’t join her class online because it’s not for where she’s at, so that meant me homeschooling while she was here. That was a big challenge.”*

The peer support work role may require difficult emotional labour, and PSWs deal with the difficulties in various ways. It is well known that PSWs need to exercise self-care and that this takes different forms for different individuals [13, 27]. Intersecting home and work roles made it difficult for some PSWs to recover from emotional and complicated peer support sessions. A PSW told us about how exercising different roles simultaneously was challenging and left some mental health needs unsatisfied:

*“When school was closed, my child was at home. Normally when I would go to work, my child is at school, I’m at work and for those six hours, I’m not a parent, I’m doing peer support … If I had a difficult group, maybe something I felt was difficult or maybe a difficult phone call, I can’t just cry in the other room [at home] because there’s homework or virtual school. For me, that’s been really challenging because I don’t feel like I have a separation between my work and my personal life, which I feel that I need for my own mental health.”*

As the above indicates, PSWs experienced challenges associated with an overlap of work and home tasks. In the next section, we consider PSWs’ boundary management tactics.

### Work-home boundary management

The above findings show that the early stage of the pandemic was a new experience for PSWs and navigating working from home posed many challenges. As the pandemic progressed, and work from home persisted, PSWs implemented tactics to protect their personal and work roles by identifying their boundaries and managing them. These boundary management tactics were individualized in the sense that each PSW had specific contingencies they needed to take into consideration in managing boundaries (such as the availability of space in the home, and the timing of other family members’ needs). The process by which the PSWs developed boundary management tactics was gradual and somewhat experimental. It took shape as PSWs gained experience and learned about solutions that worked for them:

*[At first,] I couldn't figure out how to make it work, … how to keep it separate … . And one day it occurred to me that I could make an office. … I saw somebody's home office in one of our staff meetings … . I have a large master bedroom, … so I thought, oh, I'll just put a little desk in there. And so half my room is an office, and half is my bed and whatever. And I really try to keep them separate.” learn*

*“I share my house with my [partner], and they also have virtual work to do … We had to buy another computer because we were trying to organize when someone was on the computer, and it wasn’t gonna work. We also shared an office, and we very immediately realized that it wasn’t gonna be possible … There was adapting in our home.”*

These quotes show that PSWs had to adapt to virtual work from home and implement temporal and physical changes that allowed them to navigate the work and home boundaries, and that doing so was not an easy endeavour. Yet they found tactics that allowed them to manage boundaries. Most tactics involved segmentation (or separation) of the domains as in the above two quotes, but not all tactics did. In the remainder of this section, we elaborate on the work-home boundary management strategies that PSWs implemented. We categorized them into *segmenting* tactics involving separating the domains and *integrating* tactics involving allowing some permeability and integration of the domains. Each of these boundary management strategies is further broken into tactics as shown in Table 1. Note that these tactics were not mutually exclusive, as it was possible for an individual to separate time and space at once, for example, or to use negotiation not only to manage temporal boundary challenges but also physical challenges. We separate them here to facilitate the presentation.

### Segmenting work and non-work time

PSWs engaged in negotiations with people at work and/or home to set work-life temporal distinctions. Some PSWs had to come to agreements with managers on work schedules that took into account other important contingencies in their lives. Increased workload during the pandemic, family obligations and the need for self-care time were some of the reasons for negotiations around work schedules. A common impetus for negotiations was the needs of household members which interfered with work hours during the pandemic, especially given the lockdowns. Adjusting the work schedules became necessary for PSWs who were parents of young children needing to be home-schooled or parents needing to be present with their children during virtual classes.

*“I went to my manager and said, ‘I’m going to be no good to any of you and I’m not gonna be able to carry my weight if I don’t reduce my workload … ’. They [the managers] honoured that and I went down to [x] hours a week, and I helped out when I could until I felt better, basically until [my child’s] school ended. That definitely helped because it gave me time to do some self-care. It gave me time to do those pieces because I couldn’t control these other things, and I’m not gonna go dump my [child] somewhere, or let [their] needs not to be met.”*

*“[My child] couldn’t join the class online, so that meant me homeschooling. That was a big challenge … I worked my hours when [my child] was either in bed or at [other family member’s] … . Work was flexible and adjusted to what I needed because otherwise I just couldn’t work*.

Some PSWs set out actively to limit time spent on work activities, exercising autonomy in doing so, and setting their own rules: *“I’ve been really trying to stick to my time parameters. I work morning to afternoon … . Outside of this … I’m not working. Because that would be honoured in person.”*

PSWs also communicated with family members, attempting to create a time to do work from home – time that would be protected from other home activities. They negotiated with partners attempting to find workarounds for each other’s activities:

*“I give my [partner] my schedule, ‘at these times during the day I am not available..., you have to do whatever needs to be done when that happens … And my partner does the same thing with me, it’s a give and take. So I’m constantly protecting my personal life and constantly protecting my professional life.”*

*“We actually have home meetings to outline things like ‘when do you need our landline and is it okay?’ We actually have calendars, and we figure out when we’re in, or ‘if you want to vacuum, you have to bring that up at the meeting – like, I’m gonna vacuum today, I’m gonna be making a lot of noise, when’s the best time to do that?’ That sounds crazy, but that’s what it took for us to figure it out.”*

These temporal segmentation practices allowed PSWs to dedicate some distinct time slots to work and others to non-work activities in such a way that life contingencies that changed dramatically during the pandemic could be attended to.

### Segmenting work and non-work physical space at home

Separation of workspace from non-work space at home involved reorganizing rooms, furniture, equipment and objects, some of which had utility, for example, for home and others were symbolic of work and their presence in the non-work environment evoked work situations. The extent to which physical separation of domains could be created depended on the availability of space and resources: *“I was able to set up a spare bedroom as my office space”* and *“we got to take any of the equipment from offices home that we needed”.* When space resources were more limited, PSWs created physical segmentation through other means that were often more temporary:

*“We don’t have an extra room that we can make into an office. The computer is in the main part of my house, so if I’m doing peer support work, I put the virtual background … . If my kids are home, I don’t do the peer group work on the main computer. I have to go to the basement or to my bedroom with the laptop where I can close the door so that it’s confidential.”*

The tactics PSWs used were intended to carve out space that reduced boundary permeability. There was a strong effort to prevent home-to-work spillover, and to protect one’s private space:

*“When I’m working with peers, I try to always sit at my dining room table and that’s my peer space … I’ve been considering my dining room table my office space, so when I’m with peers I try very much to stay in that space. I’m trying to keep this [other] part of my house private.”*

For most PSWs, the work and non-workspace areas were signified by different furniture, equipment and other objects that PSWs used for the accomplishment of work tasks. For PSWs with personal interests requiring the use of computers or laptops (e.g. video games), the use of different items of equipment enabled the PSWs to prevent their peer support work from blending with their other interests or activities: *“I made a decision to get this other laptop so that when I'm working, I open the work laptop and that seems to be really helping. So then when I close it, I'm done.”* Other objects were also used to create segmentation, sometimes symbolically because the objects would have utility in both the work and home spaces, yet designating a specific object for one space (e.g. work) only signalled the entry of the PSW to that (work) space:

*“What I do is I have a cup from work, a mug, and I only use it when I'm working.”*

*“I really like sticky notes, but I only use the green ones for work and the other ones are for my other stuff.”*

PSWs also used physical boundary markings (objects, open or closed doors) as a way to signal to other household members such as partners and children whether the members were welcome in the PSW workspace. Some PSWs indicated that if their door was shut, the message to others in the household was that they were expected not to enter the room:

*“We set a rule [with family], when the door is closed, don't come in. If the door is open the whole way, hello, anytime you want, if the door is halfway closed, then I'm working, but if you need to, then you can come in.”*

The physical borders that PSWs created allowed them to segment the work and non-work domains. This segmentation allowed them to detach from work when they exited the workspace or distanced themselves from work objects. Leaving the work-related physical markings behind allowed the psychological transition to the non-work space.

### Integrating domains partially - opening boundaries

Most of the tactics that PSWs told us about were those intended to create segmentation and separation of the work and home domains, and we have addressed those in the previous two sections. However, there were a few instances and tactics of partially integrating domains and allowing some boundary permeability. A few PSWs told us that they allowed some boundary permeability and blurring of the domains either because they had previous experiences setting boundaries, felt the need to help others or were personally comfortable with opening aspects of personal life (e.g. pets) to others. The following quote is from a PSW who indicated that they may receive calls during personal time, but that they would tell the caller how much time is available for the call, at the end of which the PSW terminates the call:

*“My boundaries are very wide and liberal … I’ve been doing the work that I do for [many] years, so setting boundaries with people comes a little easier for me. I tell people they can call me “whenever”, however, I also tell them that I may not pick up the phone. If I do pick up the phone, then I let people know that I only have “x minutes” and when “x” is done then I thank them and am able to end the call.”*

On the subject of opening temporal boundaries and taking work calls during personal time, another PSW mentioned a sense of responsibility towards colleagues, especially in the context of the pandemic and the difficulties that it had created for many coworkers:

*“I don’t have [many family responsibilities], so I’ve put that pressure on myself knowing that a lot of my coworkers have school-aged children, and that, in particular, challenges during COVID have been extremely stressful. I’ve been taking on a lot of stuff … It’s also just my general boundaries with myself, I’ve kind of loosened them a little bit. And that’s not been super healthy for me either, but I’m still doing it … I'm like, we need to support our staff to maintain their wellness … (The staff) know if there's something they can't deal with or they're struggling with … , I'll pick up my phone if I'm available.”*

Some PSWs also stated that they had allowed some degree of flexibility and willingness to accept spillover from home to work when it had a positive impact on their work and no negative impact on their personal lives. One topic that some PSWs did not mind sharing with others in work situations was their pets:

*“I have my dog and he barks, so people [on the phone] will be like “hey what’s your dog’s name, how long have you had your dog?” That kind of stuff is okay because I’m used to going into it. People often see pictures of my dog at work, because my dog is very cute so I’ll show pictures to make people happy, it works pretty well. So that’s kind of all stuff I’m used to talking about anyways.”*

The quotes in this section show that there were instances when PSWs either were comfortable with some boundary permeability or felt a sense of responsibility to co-workers to be available to them during personal time. However, as the second quote in this section shows, allowing boundary permeability could alleviate some work challenges, but create other challenges for the PSW.

The quotes in this and previous sections also point to several factors that influenced the boundary tactics that PSWs utilized. We had indicated earlier that these tactics tend to be idiosyncratic to individuals and depend on a number of contingencies. Although the influences were not the subject of this study, the data we collected provided indication of some of these influences. These included the personal preferences of the individuals (e.g*. “my boundaries are wide and liberal”*), the non-work obligations and roles occupied by the individuals such as being a parent, a partner (e.g. “*I’m not gonna go dump my [child] somewhere”*), the resources provided by the organization (e.g. “*we got to take any of the equipment from offices home that we needed”*), and the understanding of the managers of the individual difficulties that PSWs were facing and the support managers provided (e.g. *“[The managers] honoured that and I went down to [x] hours a week”).*

In the next section, we discuss our findings in light of the literature.

## Discussion

### Boundary challenges and management

The COVID-19 pandemic had an unprecedented impact on the work-life boundaries of health workers who had to quickly pivot to providing services remotely. In this study, we focused on PSWs whose roles had to transition rapidly from in-person to virtual service provision from home. Research shows that role change tends to occur over an extended period of time during which various adjustments are made allowing health care workers to adapt to the change [50, 51]. However, the pandemic and mandated lockdowns required immediate role changes, giving PSWs limited time to adjust. The sudden change created uncertainty and confusion [52], prompting the workers to navigate a chaotic situation fraught with temporal, physical and task boundary challenges.

Our findings reveal some similarities with those of other studies. In a scoping review, Chemali et al [53] indicate that most research shows that the effects of the pandemic on health care workers were negative. The studies they reviewed document strain, increased workload, disrupted work-life boundaries, and work-life imbalance (e.g. [7, 54]). Similar findings were reported in an empirical study by Rapp et al [37]. However, these studies have tended to focus on physicians and nurses, with limited attention to individuals from other occupations. A study that focused on the main themes of discussion that came up among social workers participating in mutual support groups during the pandemic reported that one of these themes related to challenges associated with balancing time for professional and personal life [36]. Social workers reported challenges in maintaining a healthy work–life balance and pointed to stress and difficulty maintaining professional–personal life boundaries, especially during the first two months of the pandemic. Increasing levels of stress and exhaustion were due to removing the rigidity related to particular work settings and hours. Together, these studies focus on the effects of the pandemic more generally, and not specifically on types of boundaries in the case of work-from-home situations.

Our study of PSWs showed that some participants experienced work-life temporal boundary challenges associated with work schedule encroachments and continuous online presence. Extended work availability is shown to adversely impact employees’ overall well-being and to be associated with emotional exhaustion [55]. We also found that integration of work and home domains can create physical boundary challenges, a commonly identified boundary issue in remote work/work-from-home literature [56]. In our study, the physical boundary challenges manifested as limited workspace segregation from home space, and co-presence of household members in the workspace. Working from home also created challenges for PSWs in terms of managing task boundaries. PSWs’ work, by definition, involves providing support, and this becomes ingrained in the role and identity of the workers. We found that PSWs juggled the tasks associated with meeting the needs of peers (clients), co-workers who also needed support, and family members who needed attention. This created dilemmas regarding how participants needed to direct their support tasks, energy and resources.

Our study also revealed the PSWs to be resourceful and capable of managing the challenges. Despite an initial period of difficulty when the changes were first enacted, PSWs identified their boundaries and sought tactics to actively manage them. The tactics involved segmentation or integration of work-life domains. Some of these strategies have been documented in studies of other occupations (e.g. Kreiner et al [40]), but as far as we know, no study has provided an in-depth view of PSWs’ or mental health workers’ work-home boundary management tactics when engaging in virtual work.

Our findings showed that some PSWs strategically sought a degree of boundary integration that allowed controlled permeation between work and home domains. Thus, workers took calls out of work hours and selectively addressed home-related topics (e.g. pets) in the context of work activities. However, integration was not the only way work-life boundaries were managed, and in fact, it was sought less frequently than segmentation of work and home domains. Segmentation implies closing the boundaries, which protects a domain from incursions by other domains [57]. Thus, we saw PSWs negotiating with others and finding workarounds, actively limiting time spent on work activities, designating separate physical spaces for work and non-work activities, and using physical markings and objects to symbolically separate the domains. Research shows that physical and mental separation from work enables individuals to disengage and recover from work-related responsibilities [44].

Previous research acknowledges variations in tactics that individuals seek and considers idiosyncrasies and preferences in the degree of integration of work and nonwork roles [40, 58]. The role of an employee as an active agent in the construction of boundaries has been highlighted in the work-home/life literature [24, 40]. In our study, PSWs’ boundary management tactics depended on various factors such as their personal preferences, the support they received from managers, their life commitments, and the physical space that was available to them. Not everyone chose the same degree of boundary permeability, implying that how work-life balance is achieved varies by individuals. In a scoping review of virtual work from home during the pandemic, Elbaz et al ([59], p.1) identified “heterogenous findings … with regard to work–life balance and psychological health” and the inconsistencies appeared to depend on the frequency of telework, presence of children in the home, and individual boundary management strategies among other factors.

### Strengths, implications, limitations and future research

Our study contributes to the literature by advancing the understanding of PSWs’ work-life boundary challenges and boundary management tactics in the context of work-from-home. This is an important topic given a) increasing needs for mental health services and support and b) expectations that virtual work will become more common in future years, and that in many cases, this work may be performed in employees’ homes. Work-life boundary challenges and management are critical matters for PSWs because work in the area of peer support entails interacting with people struggling with or recovering from mental illness, and PSWs themselves have had lived experience of mental health challenges that could be triggered during peer support work [60]. This study contributes to the understanding of mental health workforce challenges and potential solutions associated with work-life boundaries.

Our study contributes to practice by informing mental health workers about the strategies they can use to manage work-life boundaries. It also contributes by highlighting important considerations for human resource management decisions and policies. These policies could reflect flexible and more employee-oriented arrangements when a transition to work from home is required. Organizations can promote employee participation in decision-making processes that affect their work-life boundaries. Instituting flexible and variable work arrangements that take into consideration diversity of needs among employees can go a long way, especially in a context where turnover has been extensive in health workforce. Providing training related to the management of work-life boundaries such as mindfulness and learning to work “smarter” through time management [61, 62] can also be beneficial.

Our study has limitations. We collected our primary data in earlier stages of the pandemic, and as our post-lockdown material indicates, participants’ challenges and boundary management tactics may change as they became more experienced with virtual work from home. Future longitudinal research can be conducted to capture how boundary challenges and boundary management strategies change with time. A longitudinal study would also be able to capture the challenges and opportunities of a reverse shift to in-person service provision. Further, given that this is a qualitative study based on data from participants in one organization, the results cannot be generalized widely. However, we have provided information on the context (unexpected, mandated and rapid changes) and deep descriptions of the PSWs’ living conditions and experiences that would allow transferability of the findings to other similar contexts [47]. In addition, we pointed out that the majority of the PSWs who participated in the study identified as female. The role of gender in the navigation of work and nonwork role reconfiguration was not addressed in our study. Other research has found that gender dynamics can impact the experience of work-life boundaries and balance [23, 57]. Future research is needed on gendered experiences of work-home boundaries of mental health workers and on their boundary management strategies [63].

## Conclusion

This study shows that the sudden transition to work-from-home during the COVID-19 pandemic has had a considerable impact on PSWs' work-life boundaries. PSWs’ experiences consisted mostly of challenges associated with temporal, physical and task boundaries. PSWs demonstrated resourcefulness and adaptability to work-from-home mandates by segmenting and integrating domains. We strongly urge attention to the consequences of greater work-life integration for PSWs as well as other mental health workers since their successful practice is largely dependent on maintaining self-care.

## Data Availability

The dataset used in this research is not publicly available as set out by the research ethics approval from the University of Ottawa and the consent forms signed by the participants. Further information is available from the corresponding author upon request.

## Abbreviations

PSW: Peer support worker
UK: United Kingdom
US: United States
